# In-hospital outcomes of transcatheter aortic valve replacement in patients with chronic and end-stage renal disease: a nationwide database study

**DOI:** 10.1186/s12872-023-03684-z

**Published:** 2024-01-03

**Authors:** Marta Lorente-Ros, Subrat Das, Aaqib Malik, Francisco Jose Romeo, Jose S. Aguilar-Gallardo, Maya Fakhoury, Amisha Patel

**Affiliations:** 1 Department of Medicine, Icahn School of Medicine at Mount Sinai, Mount Sinai Morningside Hospital, New York, NY USA; 2 Department of Cardiology, New York Medical College, Westchester Medical Center, Valhalla, NY USA; 3 Division of Nephrology, Icahn School of Medicine at Mount Sinai, Mount Sinai Morningside Hospital, New York, NY USA; 4 Department of Cardiology, Icahn School of Medicine at Mount Sinai, Mount Sinai Morningside Hospital, New York, NY USA

**Keywords:** Transcatheter aortic valve replacement, Chronic kidney disease, End-stage renal disease, Renal insufficiency

## Abstract

**Background:**

Chronic kidney disease (CKD) and end-stage renal disease (ESRD) have been associated with worse outcomes after transcatheter aortic valve replacement (TAVR). With TAVR indications extending to a wider range of patient populations, it is important to understand the current implications of chronic renal insufficiency on clinical outcomes. We aim to determine the impact of CKD and ESRD on in-hospital outcomes after TAVR.

**Methods:**

We queried the National Inpatient Sample for TAVR performed between 2016 and 2020 using International Classification of Diseases-10th Revision codes. We compared in-hospital mortality and clinical outcomes between three groups: normal renal function, CKD and ESRD. The association between CKD/ESRD and outcomes was tested with multivariable logistic regression analyses, using normal renal function as baseline.

**Results:**

In the five-year study period, 279,195 patients underwent TAVR (mean age 78.9 ± 8.5 years, 44.4% female). Of all patients, 67.1% had normal renal function, 29.2% had CKD, and 3.7% had ESRD. There were significant differences in age, sex, and prevalence of comorbidities across groups. In-hospital mortality was 1.3%. Compared to patients with normal renal function, patients with renal insufficiency had higher in-hospital mortality, with the highest risk found in patients with ESRD (adjusted odds ratio: 1.4 [95% confidence interval: 1.2–1.7] for CKD; adjusted odds ratio: 2.4 [95% confidence interval: 1.8–3.3] for ESRD). Patients with CKD or ESRD had a higher risk of cardiogenic shock, need for mechanical circulatory support, and vascular access complications, compared to those with normal renal function. In addition, patients with ESRD had a higher risk of cardiac arrest and periprocedural acute myocardial infarction. The incidence of conversion to open heart surgery was 0.3% and did not differ between groups. Post-procedural infectious and respiratory complications were more common among patients with CKD or ESRD.

**Conclusion:**

Patients with CKD and ESRD are at higher risk of in-hospital mortality, cardiovascular, and non-cardiovascular complications after TAVR. The risk of complications is highest in patients with ESRD and does not result in more frequent conversion to open heart surgery. These results emphasize the importance of individualized patient selection for TAVR and procedural planning among patients with chronic renal insufficiency.

**Supplementary Information:**

The online version contains supplementary material available at 10.1186/s12872-023-03684-z.

## Background

Chronic kidney disease (CKD) is a common comorbidity in patients with aortic stenosis and an independent risk factor for mortality in those undergoing transcatheter aortic valve replacement (TAVR) [1–6]. In addition to higher short- and long-term mortality, CKD confers a higher risk of vascular access complications and major bleeding after TAVR [5, 7–9]. This risk increases with the severity of renal insufficiency [2, 6–8, 10]. Similarly, end-stage renal disease (ESRD) is associated with more procedural complications after TAVR [11, 12]. The mortality of patients with ESRD undergoing TAVR reaches 40% at one year [11, 13], which raises the challenge of refining selection of potential candidates for TAVR in this population.

Most of these observations have been made in patients at high risk for surgical aortic valve replacement (SAVR). With TAVR indications extending to intermediate [14, 15] and low surgical risk patients [16, 17], and with technical innovations in devices introduced in clinical practice, it is important to establish the current impact of pre-procedural chronic renal insufficiency on clinical outcomes of patients undergoing TAVR. The objective of this study is to determine the association of CKD and ESRD with in-hospital mortality and outcomes in patients undergoing TAVR.

## Methods

### Study design and patient population

This is an observational, retrospective, nationwide cohort study. We used the National Inpatient Sample database, an all-payer inpatient healthcare database which approximates a 20% stratified sample of all discharges from the United States community hospitals, excluding rehabilitation and long-term acute care hospitals. This database is being developed as part of the Healthcare Cost and Utilization Project and is the largest publicly available all-payer inpatient database. The data is weighted to obtain national estimates of hospital stays across the United States.

We retrospectively queried the database for the years 2016 to 2020. Hospital admissions for TAVR were identified using the International Classification of Diseases-10th Revision (ICD-10) diagnosis codes 02RF38H, 02RF38Z, 02RF48Z, 02RF3KZ. All patients aged ≥ 18 years who underwent TAVR were included. No exclusion criteria were applied. Patients with CKD were identified using ICD-10 codes N181, N182, N183, N184, N185, N189. Patients with ESRD were identified using ICD-10 code N186. CKD stage 1 (ICD-10 code N181) was defined as kidney damage with normal or increased estimated glomerular filtration rate (eGFR) of ≥ 90 mL/min. CKD stage 2 (ICD-10 code N182) was defined as kidney damage with eGFR 60–89 mL/min. CKD stage 3 (ICD-10 code N183) was defined as kidney damage with eGFR 30–59 mL/min. CKD stage 4 (ICD-10 code N184) was defined as kidney damage with eGFR 15–29 mL/min. CKD stage 5 (ICD-10 code N185) was defined as kidney damage with eGFR < 15 mL/min. CKD with unspecified stage (ICD-10 code N189) included all patients in which there was lack of data regarding staging of CKD. ESRD (ICD-10 code N186) was defined as chronic kidney damage with eGFR < 15 mL/min requiring hemodialysis or peritoneal dialysis. To determine the presence of comorbidities we used appropriate ICD-10 codes.

### Outcomes

We studied the in-hospital outcomes of all-cause mortality, length of stay, cardiac arrest, cardiogenic shock, use of mechanical circulatory support (MCS) with Impella^®^ device or intra-aortic balloon, procedural acute myocardial infarction (AMI), vascular access complications (which included intra- or post-procedural hemorrhage or hematoma requiring transfusion, vascular injury, and aneurysm), conversion to open heart surgery, post-procedural infection of any source, respiratory complications, and acute kidney injury (AKI). AKI was defined by ICD-10 codes as acute renal failure with tubular, cortical or medullary necrosis (ICD-10 code N17), post-procedural renal failure (ICD-10 code N99), or post-procedural complications of the genitourinary system (ICD-10 code N99.89). Other ICD-10 codes used to identify the incidence of outcomes are shown in Supplementary Table 1.

### Statistical analysis

Quantitative variables are presented as mean ± standard deviation of the mean (SD) and were compared using one-way analysis of variance. Qualitative categorical variables are presented as n (%) and were compared using the Pearson’s chi-square test. The association between chronic renal insufficiency and in-hospital outcomes was tested in multivariable analysis for CKD and ESRD, each compared to the group with normal renal function. Multivariable analysis was performed using logistic regression for qualitative binary outcomes and linear regression for continuous outcomes. The strength of the associations was measured by the adjusted odds ratio (aOR) and 95% confidence interval (CI). Variables included in the regression model were demographics and comorbidities that were statistically significant between groups at baseline, namely age, sex, race, atrial fibrillation, CHA_2_DS_2_-VASc score, obesity, hypertension, diabetes, hyperlipidemia, tobacco use, pulmonary hypertension, prior stroke, prior percutaneous coronary intervention, and prior coronary artery bypass graft surgery. A *p* value < 0.05 was considered statistically significant. Statistical analysis was performed using Stata 16.0 (StataCorp^®^, College Station, TX). The figure was created using GraphPad Prism.

## Results

From January 2016 to December 2020, we identified a total of 279,195 patients who underwent TAVR in the United States. The mean age ± SD of the population was 78.9 ± 8.5 years. 44% of the patients were female and 84.6% were white. CKD was present in 81,640 patients (29.2%) and ESRD in 10,230 patients (3.7%). The remaining patients (67.1%) had normal renal function at baseline. From the patients with CKD, 0.7% had CKD stage 1, 7.7% had CKD stage 2, 56.2% had CKD stage 3, 11.7% had CKD stage 4, 10.4% had CKD stage 5, and 13.3% had CKD of unspecified stage. The majority of TAVR procedures (89.4%) were done in teaching hospitals (Table 1).

Of all patients, 66.2% had heart failure, 37.3% had atrial fibrillation, 21.9% had a prior percutaneous coronary intervention, and 7.3% had peripheral arterial disease. Diabetes, hypertension, and hyperlipidemia were present in 37.7%, 89.8%, and 72.3% of patients, respectively. There were significant differences in age, sex, race, and prevalence of comorbidities across groups (Table 1).

**Table 1:** Baseline characteristics of patients undergoing TAVR in the United States from 2016 to 2020

	All patients n = 279,195	Normal renal function n = 187,325	CKD n = 81,640	ESRD n = 10,230	*p* value
Age, yrs, mean (SD)	78.9 (8.5)	78.5 (8.6)	80.4 (7.7)	72.2 (10.0)	< 0.001
Female sex, %	44.4	46.9	39.8	35.9	< 0.001
Race, %					
White	84.6	86.1	83.7	64.0	< 0.001
Black	4.0	3.0	4.6	17.4	< 0.001
Hispanic	4.6	4.3	4.6	9.4	< 0.001
Other	3.7	3.6	3.7	6.5	< 0.001
Missing data	3.1	3.0	3.4	2.7	
**Comorbidities**					
Prior AMI, %	12.2	10.9	14.7	15.7	< 0.001
Prior PCI, %	21.9	21.3	23.3	20.4	< 0.001
Prior CABG, %	15.4	14.4	17.9	13.0	< 0.001
PAD, %	7.3	6.7	8.5	8.2	< 0.001
Heart failure, %	66.2	61.8	75.2	75.3	< 0.001
Atrial fibrillation, %	37.3	34.9	42.9	38.3	< 0.001
CHA_2_DS_2_-VASc, mean (SD)	4.6 (1.4)	4.46 (1.5)	4.94 (1.4)	4.52 (1.5)	< 0.001
Obesity, %	20.3	19.9	21.5	18.4	< 0.001
Hypertension, %	89.8	87.1	95.1	96.4	< 0.001
Diabetes, %	37.7	32.6	46.8	58.6	< 0.001
Hyperlipidemia, %	72.3	72.3	73.6	62.0	< 0.001
Tobacco use, %	40.3	41.0	39.4	34.4	< 0.001
COPD, %	20.5	19.4	23.1	21.2	< 0.001
Pulmonary hypertension, %	15.6	14.1	19.0	24.6	< 0.001
Prior stroke, %	13.5	12.9	14.7	14.2	< 0.001
Prior TIA, %	11.6	11.1	12.8	11.3	< 0.001
Bicuspid valve, %	1.8	2.2	1.2	1.0	< 0.001
**Hospital type**, %					< 0.05
Teaching	89.4	89.2	89.9	90.7	
Non-teaching	10.6	10.8	10.1	9.3	

Abbreviations: AMI acute myocardial infarction, CABG coronary artery bypass grafting, CKD chronic kidney disease, COPD chronic obstructive pulmonary disease, ESRD end-stage renal disease, PAD peripheral arterial disease, PCI percutaneous coronary intervention, SD standard deviation of the mean, TAVR Transcatheter aortic valve replacement, TIA transient ischemic attack, Yrs years.

Over the five-year study period, 3,710 patients (1.3%) died in the hospital. All-cause in-hospital mortality was 1.1% in patients with normal renal function, 1.6% in patients with CKD, and 2.6% in patients with ESRD. After adjusting for significant differences in baseline characteristics on multivariable logistic regression, there was an increase in the odds of in-hospital mortality in patients with CKD or ESRD, which was highest in the group of patients with ESRD (aOR: 1.4 [95% CI: 1.2–1.7] for CKD; aOR: 2.4 [95% CI: 1.8–3.3] for ESRD) (Table 2).

Of all patients, 0.2% had cardiac arrest, 1.4% developed cardiogenic shock, and 3.1% had periprocedural AMI. MCS was used in 0.7% of patients (0.3% with Impella^®^ device and 0.4% with intra-aortic balloon). The incidence of vascular access complications in the form of hemorrhage or hematoma requiring transfusion, vascular injury, or aneurysm was 3.7%. Nine hundred and sixty patients (0.3%) required conversion to open heart surgery (Table 2). The mean length of hospital stay was 3.8 days for all patients. Compared to patients with normal renal function, those with CKD or ESRD had significantly longer hospitalizations (Table 2).

**Table 2:** In-hospital outcomes of patients undergoing TAVR in the United States from 2016 to 2020

	All patients n = 279,195	Normal renal function n = 187,325	CKD n = 81,640	ESRD n = 10,230	CKD		ESRD		
					aOR (95%CI)	*p* value	aOR (95% CI)	*p* value	
In-hospital mortality	3710 (1.3%)	2145 (1.1%)	1295 (1.6%)	270 (2.6%)	1.4 (1.2–1.7)	< 0.001	2.4 (1.8–3.3)	< 0.001	
LOS, days (SEM)	3.8 (0.04)	3.3 (0.03)	4.6 (0.06)	6.9 (0.22)	N/A	< 0.001	N/A	< 0.001	
**Cardiovascular complications**									
Cardiac arrest	620 (0.2)	340 (0.2)	230 (0.3)	50 (0.5)	1.5 (1.0-2.1)	0.058	2.4 (1.2–4.9)	0.015	
Cardiogenic shock	4010 (1.4)	2415 (1.3)	1305 (1.6)	290 (2.8)	1.2 (1.1–1.4)	0.009	2.0 (1.5–2.7)	< 0.001	
Impella	880 (0.3)	450 (0.2)	355 (0.4)	75 (0.7)	1.8 (1.3–2.5)	< 0.001	1.9 (1.0-3.4)	0.035	
Intraaortic balloon	1195 (0.4)	675 (0.4)	385 (0.5)	135 (1.3)	1.3 (0.9–1.7)	0.126	2.1 (1.3–3.3)	0.002	
Procedural AMI	8655 (3.1)	5445 (2.9)	2645 (3.2)	565 (5.5)	1.1 (0.9–1.2)	0.329	1.7 (1.4–2.1)	< 0.001	
Vascular access complications	10,235 (3.7)	6465 (3.5)	3300 (4.0)	470 (4.6)	1.2 (1.1–1.3)	0.003	1.3 (1.0-1.6)	0.043	
Conversion to open heart surgery	960 (0.3)	675 (0.4)	240 (0.3)	45 (0.4)	0.9 (0.6–1.2)	0.384	1.3 (0.6–2.6)	0.479	
**Other complications**									
Post-operative infection	2350 (0.8)	1175 (0.6)	885 (1.1)	290 (2.8)	1.8 (1.5–2.2)	< 0.001	3.0 (2.2–4.1)	< 0.001	
Respiratory complications	18,905 (6.8)	10,355 (5.5)	7010 (8.6)	1540 (15.1)	1.5 (1.4–1.6)	< 0.001	2.3 (2.0-2.6)	< 0.001	
AKI	26,770 (9.6)	9340 (5.0)	17,340 (21.2)	N/A	5.0 (4.7–5.3)	< 0.001	N/A	N/A	

Abbreviations: AKI acute kidney injury, AMI acute myocardial infarction, aOR adjusted odds ratio, CI confidence interval, CKD chronic kidney disease, ESRD end stage renal disease, LOS length of stay, TAVR transcatheter aortic valve replacement, SEM standard error of the mean.

**Fig. 1:**
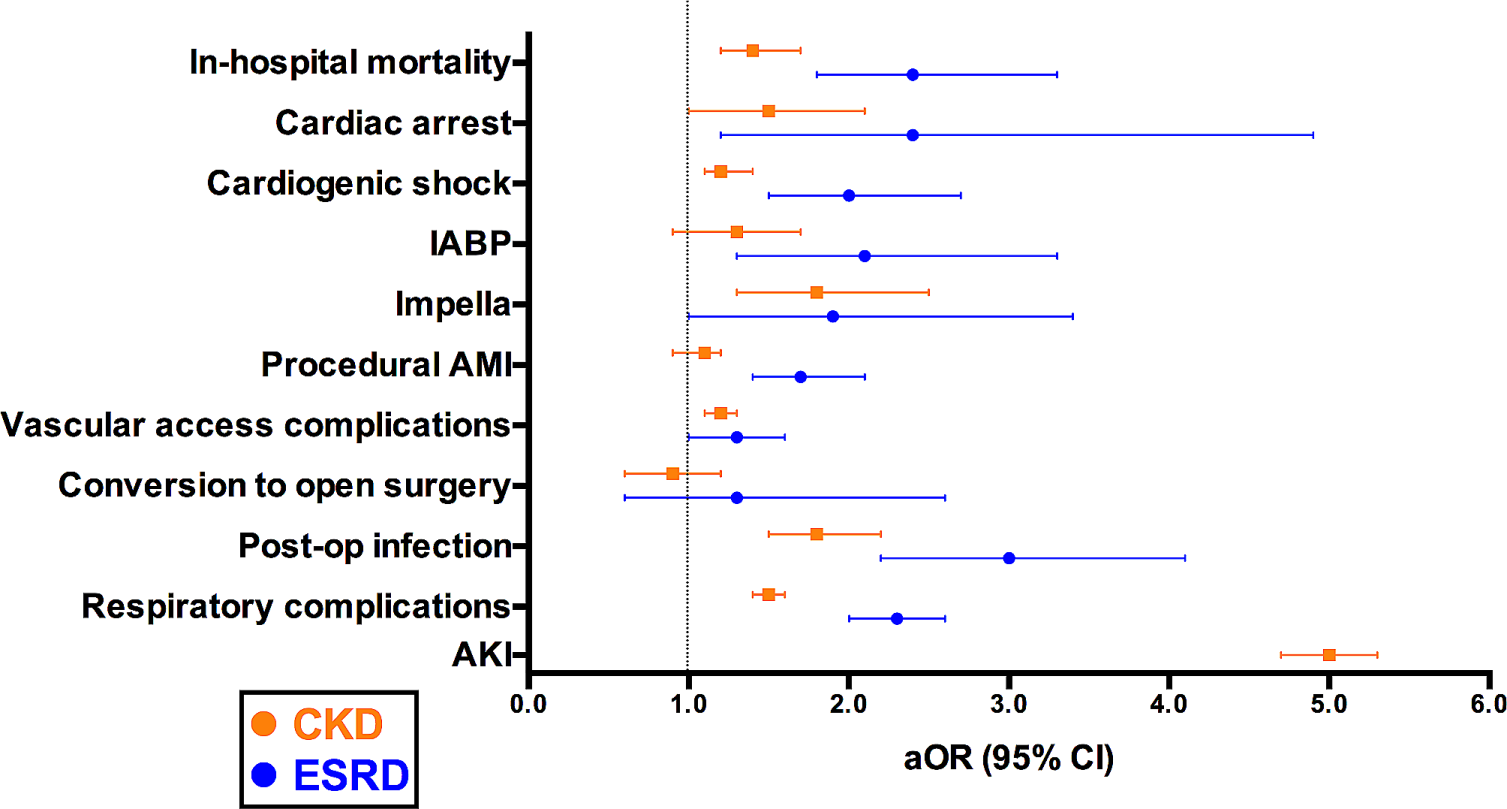
Adjusted odds ratio of in-hospital outcomes in patients with CKD and ESRD undergoing TAVR

Figure 1 represents the odds ratio of in-hospital complications in patients with CKD and ESRD, as compared to patients with normal renal function, after adjusting for differences in baseline characteristics. Patients with CKD had a higher risk of cardiogenic shock, need for MCS with Impella^®^ device, and vascular access complications (all *p* values < 0.001) (Table 2; Fig. 1). Patients with ESRD had a 2-fold increased risk of cardiogenic shock and need for any form of MCS, as well as a higher risk of vascular access complications, cardiac arrest, and procedural AMI (all *p* values < 0.001). The risk of conversion to open heart surgery was 0.3% in patients with CKD and 0.4% in patients with ESRD, with no significant difference when compared to patients with normal renal function (Table 2; Fig. 1). Within patients with ESRD, there were no significant differences between hemodialysis and peritoneal dialysis (Supplementary Table 2, Supplementary Table 3).

Post-procedural infection was more frequent in patients with CKD or ESRD, compared to those with normal renal function (*p* < 0.001). The incidence of endocarditis was 0.2% in patients with normal renal function, 0.3% in patients with CKD, and 0.3% in patients with ESRD (p = 0.822). Both patients with CKD and ESRD had a higher incidence of respiratory complications than patients with normal renal function (*p* < 0.001). The incidence of AKI during hospitalization was 5.0% in patients with normal renal function, and 21.2% in patients with CKD (aOR: 5.0 [95% CI: 4.7–5.3]) (Table 2; Fig. 1). Within patients with CKD, we compared baseline characteristics and outcomes between patients developing AKI and patients without AKI (Supplementary Table 4, Supplementary Table 5). Patients with CKD who developed AKI were at higher risk of in-hospital mortality, cardiovascular, infectious, and respiratory complications, when compared to patients with CKD who did not develop AKI (Supplementary Table 5).

## Discussion

The present study is the largest to describe the impact of chronic renal insufficiency on in-hospital outcomes after TAVR in a stratified sample of admissions in the United States. Compared to patients with normal renal function, our results show a higher risk of in-hospital mortality, cardiac complications requiring MCS, and vascular access complications in patients with CKD or ESRD, albeit with a similar incidence of conversion to open heart surgery. Patients with renal insufficiency also had longer hospitalizations and were at higher risk for post-procedural AKI, infectious and respiratory complications. The observed risk of complications was highest in patients with ESRD.

Chronic renal insufficiency increases the risk of aortic stenosis and leads to accelerated dystrophy of the aortic leaflets as a consequence of various mechanisms, including alteration in calcium-phosphate homeostasis, pathological expression of bone-related proteins, endothelial damage, and chronic inflammation [18–20]. The proportion of patients with renal insufficiency undergoing TAVR has grown from the initial PARTNER and CoreValve™ clinical trials, which included 11.1% of patients with serum creatinine > 2 mg/dL and 12.2% of patients with CKD stages 4–5, respectively [21, 22]. Both investigations excluded patients with ESRD on dialysis [21, 22]. Our study shows a higher representation of patients with CKD (29.2%) and ESRD (3.7%), consistent with prior published data from nationwide registries [11, 12, 23, 24]. Patients with CKD or ESRD are a more comorbid patient population, as our results corroborate [11, 12, 23–25]. With the growing representation of these patients in the TAVR population, it is increasingly relevant to understand the current implications of kidney disease on procedural outcomes, as it may allow for optimization of patient selection, pre-procedural risk stratification and procedural planning.

In-hospital mortality in the overall cohort of this study was low (1.3%), similar to that reported in recent years of the Transcatheter Valve Therapy Registry [26]. Patients with renal insufficiency had a higher risk of in-hospital mortality, with an aOR of 1.4 and 2.4 for CKD and ESRD, respectively. These results are in line with those of nationwide studies conducted in prior years from 2011 to 2014, which showed a 1.3 to 1.4-fold risk of mortality in patients with CKD and a 2.4 to 2.6-fold risk in those with ESRD [12, 23]. Despite a similar relative increase in risk, the incident mortality in our contemporary population was considerably lower than in prior studies (1.6% in patients with CKD, compared to 3.8–4.5% in prior studies; 2.6% in patients with ESRD, compared to 8.2–8.3% in prior studies), which could be attributed to improvement in device technology, better imaging guidance during valve deployment, or increased operator experience [12, 23].

Several investigations have described advanced CKD as an independent risk factor for early and late mortality after TAVR [1–6, 24, 27, 28]. The increased mortality risk in patients with renal insufficiency has been attributed to higher surgical risk scores, poorer functional status, concomitant severe mitral or tricuspid regurgitation, and coexistence of other cardiovascular comorbidities [10, 11]. The highest mortality is found in patients with ESRD, reaching 40% at one year and almost 90% at five years [11, 13]. Despite the high mortality rates in ESRD, aortic valve replacement is still associated with better outcomes in this population, compared to conservative management of aortic stenosis [29]. Therefore, it becomes essential to identify factors associated with survival among patients with ESRD to optimize patient selection based on the individual likelihood of procedural and functional benefit. For this purpose, Szerlip et al. suggest considering factors such as concomitant mitral and tricuspid regurgitation, duration and type of dialysis, frailty, and functional capacity [11]. In the United States Renal Data System, Ogami et al. identified age > 75 years, body mass index < 25 kg/m^2^, chronic obstructive pulmonary disease, diabetes, and white race as independent risk factors for five-year mortality in patients with ESRD [13]. A multidisciplinary approach with a heart-kidney team can be beneficial for making challenging treatment decisions in this population [30].

In our study, cardiac complications were more frequent in patients with renal insufficiency. Patients with CKD or ESRD had a higher risk of cardiogenic shock and need for MCS. While CKD did not confer a higher risk of other cardiac complications, patients with ESRD more frequently had cardiac arrest and periprocedural AMI, compared to patients with normal renal function. Notably, this higher incidence of peri-procedural cardiac events did not translate into more frequent conversion to SAVR. Prior studies with smaller sample sizes did not evaluate the outcomes of cardiac arrest, cardiogenic shock, or use of MCS. Results have been inconsistent in the endpoint of conversion to SAVR, with Mohananey et al. describing a similar risk of conversion in patients with normal renal function, CKD, or ESRD, while Gupta et al. reported an increased risk in patients with ESRD [12, 23].

Vascular access complications in our cohort were slightly more common in patients with CKD or ESRD (aOR 1.2 and 1.3, respectively). Prior studies have consistently reported more bleeding and vascular access complications in patients with renal insufficiency, compared to the general population [4, 5, 8, 9, 11, 12, 23, 24], including recent meta-analyses [7, 10]. These complications are a result of uremic toxins and morphologic changes in the vessel wall, which lead to dysfunction of the coagulation cascade [31]. Procedural planning with a focus on using smaller sheaths and routine closure device utilization may be necessary to minimize vascular complications in these patients.

Notably, most of the literature describing a higher incidence of mortality and bleeding complications in patients with CKD has included a majority of patients with high surgical risk scores [1–5, 24, 28, 32]. A study by Makki et al. included subgroup analysis by different surgical risks and suggested that the increased risk for both mortality and bleeding in patients with CKD was limited to the subgroup with high surgical risk [33]. Thus, it is possible that the prognostic significance of CKD in patients undergoing TAVR varies across patients with low, intermediate, and high surgical risk, and would need to be explored in each risk stratum. Moreover, few studies focus on the optimal cut-off value of eGFR to predict negative procedural outcomes. While most reports use an eGFR< 60 mL/min/1.73 m^2^, other authors suggest that a value of eGFR< 45 mL/min/1.73 m^2^ would be a better predictor of adverse outcomes in the TAVR population [28]. Most recently, staging of CKD with cystatin C-eGFR has been proposed as a more accurate predictor of negative cardiovascular outcomes after TAVR than creatinine based eGFR, given the influence of muscle mass in creatinine eGFR [34], which would be of especial relevance in the risk stratification of sarcopenic elderly patients.

Post-procedural infectious and respiratory complications were also more common among patients with CKD or ESRD in this nationwide population, which could be related to the relative immunodepression linked to renal insufficiency [35]. Furthermore, patients with CKD had a 5-fold risk of developing AKI after TAVR, as has been described in other cohorts [24, 25]. Kidney injury after TAVR is by itself associated with an increase in one-year mortality and longer hospitalizations [36, 37], with a recent study estimating that AKI is responsible for one fifth of the negative effect of CKD on mortality after TAVR [38]. Factors that can contribute to AKI are nephrotoxicity of contrast medium, blood transfusions, renal hypoperfusion during rapid ventricular pacing, and microembolization of cholesterol plaque into the renal vasculature with vascular instrumentation [25, 37]. Risk stratification of patients based on eGFR could help prevent this complication by focusing on pre-procedural volume optimization and limitation of contrast load, with emerging evidence of alternate imaging modalities to limit or avoid iodinated contrast [39–42]. To date, the optimal choice of anesthetic strategy for renal protection during TAVR remains unclear. Despite the increasing use of conscious sedation [43], it has not been found to lower the risk of AKI as compared to a general anesthesia approach [44, 45]. Finally, the promising early results of the RenalGuard system (PLC Medical Systems), which matches infusion of isotonic saline to furosemide-induced diuresis, have not been confirmed in subsequent studies and therefore it is not currently validated as a nephroprotective strategy during TAVR [46, 47].

### Limitations

Despite the large sample size and representative nature of TAVR admissions in the United States, there are inherent limitations to this study. Firstly, given its retrospective nature, conclusions are limited to in-hospital outcomes. Secondly, intrinsic limitations to the database of the National Inpatient Sample, an administrative, claim-based, database, precluded the study of clinically relevant outcomes, such as post-procedural embolic stroke and permanent pacemaker implantation. Pertinent peri-procedural data such as TAVR access site (endovascular vs. transapical), or whether vascular access complications were related to access site or MCS, was also not accounted for. Due to the absence of laboratory values in the database, the accuracy of the definition of AKI as well as the staging of CKD should be interpreted with caution. Thirdly, the database does not include information on the specific indication for each TAVR, or the type of device used for each patient. Finally, this study does not include stratification of patients by surgical risk, and it is possible that the impact of renal insufficiency on clinical outcomes after TAVR varies across different surgical risks.

## Conclusions

In conclusion, a higher risk of in-hospital mortality persists in patients with CKD and ESRD undergoing TAVR, despite inclusion of lower surgical risk patients, increased operative experience, technological advancements in transcatheter heart valves, and an overall lower incidence of mortality events. Moreover, renal insufficiency is associated with a higher risk of cardiovascular, respiratory, infectious, and renal complications after TAVR. The risk of complications is highest in patients with ESRD and does not seem to result in a higher risk of conversion to open heart surgery. These results emphasize the importance of multidisciplinary, individualized, patient selection for TAVR among those with chronic renal insufficiency. A focus on pre-procedural optimization strategies and procedural planning may be necessary to minimize the risk of complications in this patient population.

### Electronic supplementary material

Below is the link to the electronic supplementary material.

Supplementary Material 1

## Data Availability

The dataset used and analysed in the current study is a publicly available dataset (National Inpatient Sample), part of the Healthcare Cost and Utilization Project from the United States, and can be accessed at the following link: https://hcup-us.ahrq.gov/nisoverview.jsp.

## Abbreviations

AKI: Acute kidney injury
AMI: Acute myocardial infarction
aOR: Adjusted odds ratio
CI: Confidence interval
CKD: Chronic kidney disease
eGFR: Estimated glomerular filtration rate
ESRD: End-stage renal disease
ICD-10: International Classification of Diseases-10th Revision
MCS: Mechanical circulatory support
SAVR: Surgical aortic valve replacement
SD: Standard deviation of the mean
TAVR: Transcatheter aortic valve replacement
