# Supplementary material for: In-hospital outcomes of transcatheter aortic valve replacement in patients with chronic and end-stage renal disease: a nationwide database study
Source: BMC Cardiovasc Disord. 2024 Jan 3;24:21. doi: 10.1186/s12872-023-03684-z (PMC10765730; doi:10.1186/s12872-023-03684-z)
Supplement: Supplementary file 1 — Supplementary Material 1 [file 12872_2023_3684_MOESM1_ESM.docx]

**In-Hospital Outcomes of Transcatheter Aortic Valve Replacement in Patients with Chronic and End-Stage Renal Disease: A Nationwide Database Study**

**Supplementary material**

Supplementary Table 1: ICD-10 codes used to define outcomes. Page 2.

Supplementary Table 2: Baseline characteristics of patients with ESRD on peritoneal dialysis or hemodialysis. Page 3.

Supplementary Table 3: In-hospital outcomes of patients with ESRD on peritoneal dialysis or hemodialysis. Page 4.

Supplementary Table 4: Baseline characteristics of patients with CKD with and without acute kidney injury. Page 5.

Supplementary Table 5: In-hospital outcomes of patients with CKD with and without acute kidney injury. Page 6.

| **Supplementary Table 1: ICD-10 codes used to define outcomes.** | | |
| --- | --- | --- |
| **Variable** | | **ICD-10 code** |
| Cardiac arrest | | I9712 |
| Shock | | T8111XA, T8110XA, I977 |
| Impella | | 5A0221D |
| Intraaortic balloon | | 5A02210 |
| Procedural acute myocardial infarction | | 0270, 0271, 0272, 0273 |
| Vascular access complications | |  |
|  | Intraoperative or postoperative hemorrhage or hematoma requiring transfusion | K916, K9184, G975, I974, I97618, 30233H, 30233N |
|  | Vascular injury requiring surgical intervention | 03Q, 0W3, 04Q, 02U, 03U, 04U, 05U, 06U |
|  | Vascular injury | T81719A, T81718A, T81711A, T81710A, I975, I976, S750, S651 |
|  | Aneurysm/Acquired AV fistula (excluding ESRD) | I770, I724, S750, S651 |
| Conversion to open heart surgery | | 02Q, 0W3D0 |
| Post-operative infection | | T80211A, T8149XA, T8144XA, T8140XA,  T8112XA, A40, A41 |
| Respiratory complications | |  |
|  | Post-procedural respiratory failure | J9582, J9588, J9589 |
|  | Pneumothorax | J9581 |
|  | Intubation > 24 hours | 5A1935Z, 5A1945Z, 5A1955Z, 0B110F4, 0B113F4 |
|  | Acute respiratory failure | J960 |
|  | Acute and chronic respiratory failure | J962 |
| Acute kidney injury | | N17, N9989, N990 |
| Abbreviations: ICD-10 International Classification of Diseases 10^th^ Revision | | |

| **Supplementary Table 2: Baseline characteristics of patients with ESRD on peritoneal dialysis or hemodialysis.** | | | | |
| --- | --- | --- | --- | --- |
|  | **All ESRD**  n = 10,230 | **Peritoneal dialysis**  n = 535 | **Hemodialysis**  n **=**9,695 | **p value** |
| Age, yrs (SD) | 72.2 (10.0) | 71.0 (10.3) | 72.3 (10.0) | <0.001 |
| Female sex, % | 35.9 | 41.1 | 35.6 | 0.242 |
| Race, % |  |  |  | 0.064 |
| White | 64.0 | 76.9 | 65.1 |  |
| Black | 17.4 | 17.3 | 17.9 |  |
| Hispanic | 9.4 | 3.8 | 10.0 |  |
| Other/Missing data | 9.2 | 1.9 | 6.9 |  |
| **Comorbidities** |  |  |  |  |
| Prior AMI, % | 15.7 | 20.6 | 15.5 | 0.154 |
| Prior PCI, % | 20.4 | 23.4 | 20.2 | 0.425 |
| Prior CABG, % | 13.0 | 13.1 | 13.0 | 0.991 |
| PAD, % | 8.2 | 14.0 | 7.8 | 0.022 |
| Heart failure, % | 75.3 | 72.9 | 75.4 | 0.549 |
| Atrial fibrillation, % | 38.3 | 35.5 | 38.5 | 0.548 |
| CHADS-VASc, mean (SD) | 4.52 (1.5) | 4.4 (1.5) | 4.5 (1.5) | 0.463 |
| Obesity, % | 18.4 | 21.5 | 18.2 | 0.383 |
| Hypertension, % | 96.4 | 97.2 | 96.3 | 0.644 |
| Diabetes, % | 58.6 | 63.6 | 58.3 | 0.291 |
| Hyperlipidemia, % | 62.0 | 70.1 | 61.6 | 0.081 |
| Tobacco use, % | 34.4 | 22.4 | 35.1 | 0.008 |
| COPD, % | 21.2 | 18.7 | 21.4 | 0.505 |
| Pulmonary hypertension, % | 24.6 | 15.9 | 25.1 | 0.031 |
| Prior stroke, % | 14.2 | 9.3 | 14.4 | 0.139 |
| Prior TIA, % | 11.3 | 6.5 | 11.6 | 0.105 |
| **Hospital type**, % |  |  |  | 0.396 |
| Teaching | 90.7 | 92.5 | 90.6 |  |
| Non-teaching | 9.3 | 7.5 | 9.4 |  |
| Abbreviations: AMI acute myocardial infarction, CABG coronary artery bypass grafting, COPD chronic obstructive pulmonary disease, ESRD end-stage renal disease, PAD peripheral arterial disease, PCI percutaneous coronary intervention, SD standard deviation of the mean, TIA transient ischemic attack, Yrs years. | | | | |

| **Supplementary Table 3: In-hospital outcomes of patients with ESRD on peritoneal dialysis or hemodialysis.** | | | | |
| --- | --- | --- | --- | --- |
|  | **All ESRD**  n = 10,230 | **Peritoneal dialysis**  n = 535 | **Hemodialysis**  n **=**9,695 | **aOR**  **(95% CI)** |
| In-hospital mortality, % | 2.6 | 1.9 | 2.7 | 0.46  (0.13-1.62) |
| LOS, days (SEM) | 6.9 (0.22) | 5.4 (0.32) | 7.0 (0.10) | N/A |
| **Cardiovascular complications, %** | | | | |
| Cardiac arrest | 0.5 | 0.0 | 0.5 | N/A |
| Cardiogenic shock | 2.8 | 2.8 | 2.8 | 1.25  (0.36-4.28) |
| Impella | 0.7 | 0.9 | 0.7 | 1.11  (0.22-5.60) |
| Intraaortic balloon | 1.3 | 1.9 | 1.3 | 2.21  (0.47-10.34) |
| Procedural AMI | 5.5 | 5.6 | 5.5 | 1.07  (0.42-2.72) |
| Vascular access complications | 4.6 | 6.5 | 4.5 | 1.72  (0.69-4.31) |
| Conversion to open heart surgery | 0.4 | 0.0 | 0.5 | N/A |
| **Other complications, %** |  |  |  |  |
| Post-operative infection | 2.8 | 0.9 | 2.9 | 0.34  (0.04-2.73) |
| Respiratory complications | 15.1 | 11.2 | 15.3 | 0.67  (0.32-1.4) |
| Abbreviations: AKI acute kidney injury, AMI acute myocardial infarction, aOR adjusted odds ratio, CI confidence interval, CKD chronic kidney disease, LOS length of stay, SEM standard error of the mean. | | | | |

| **Supplementary Table 4: Baseline characteristics of patients with CKD with and without acute kidney injury.** | | | | |
| --- | --- | --- | --- | --- |
|  | **All CKD**  n = 81,640 | **No AKI**  n = 64,300 | **AKI**  n = 17,340 | **p value** |
| Age, yrs (SD) | 80.4 (7.7) | 80.6 (7.4) | 79.6 (8.5) | <0.001 |
| Female sex, % | 39.8 | 39.6 | 40.3 | 0.447 |
| Race, % |  |  |  | <0.001 |
| White | 83.7 | 87.8 | 82.1 |  |
| Black | 4.6 | 4.3 | 6.4 |  |
| Hispanic | 4.6 | 4.2 | 7.0 |  |
| Other/ Missing data | 7.1 | 3.7 | 4.5 |  |
| **Comorbidities** |  |  |  |  |
| Prior AMI, % | 14.7 | 15.2 | 12.8 | 0.001 |
| Prior PCI, % | 23.3 | 24.5 | 18.6 | <0.001 |
| Prior CABG, % | 17.9 | 18.2 | 16.7 | 0.034 |
| PAD, % | 8.5 | 8.1 | 9.9 | 0.001 |
| Heart failure, % | 75.2 | 72.5 | 85.2 | <0.001 |
| Atrial fibrillation, % | 42.9 | 41.6 | 48.1 | <0.001 |
| CHADS-VASc, mean (SD) | 4.9 (1.4) | 4.9 (1.3) | 5.1 (1.3) | <0.001 |
| Obesity, % | 21.5 | 21.3 | 21.3 | 0.931 |
| Hypertension, % | 95.1 | 95.0 | 95.6 | 0.175 |
| Diabetes, % | 46.8 | 45.6 | 51.5 | <0.001 |
| Hyperlipidemia, % | 73.6 | 75.2 | 67.7 | <0.001 |
| Tobacco use, % | 39.4 | 40.9 | 33.8 | <0.001 |
| COPD, % | 23.1 | 22.2 | 26.3 | <0.001 |
| Pulmonary hypertension, % | 19.0 | 17.0 | 26.4 | <0.001 |
| Prior stroke, % | 14.7 | 15.0 | 13.8 | 0.0001 |
| Prior TIA, % | 12.8 | 13.4 | 10.5 | <0.001 |
| **Hospital type**, % |  |  |  | 0.220 |
| Teaching | 89.9 | 89.7 | 90.5 |  |
| Non-teaching | 10.1 | 10.3 | 9.5 |  |
| Abbreviations: AMI acute myocardial infarction, CABG coronary artery bypass grafting, CKD chronic kidney disease, COPD chronic obstructive pulmonary disease, ESRD end-stage renal disease, PAD peripheral arterial disease, PCI percutaneous coronary intervention, SD standard deviation of the mean, TAVR Transcatheter aortic valve replacement, TIA transient ischemic attack, Yrs years. | | | | |

| **Supplementary Table 5: In-hospital outcomes of patients with CKD with and without acute kidney injury.** | | | | |
| --- | --- | --- | --- | --- |
|  | **All CKD**  n = 81,640 | **No AKI**  n = 64,300 | **AKI**  n = 17,340 | **aOR**  **(95% CI)** |
| In-hospital mortality, % | 1.6 | 0.6 | 5.4 | 8.84  (6.62-11.79) |
| LOS, days (SEM) | 4.6 (0.06) | 3.1 (0.01) | 10.3 (0.07) | 6.66  (6.37-6.95) |
| **Cardiovascular complications, %** | | | | |
| Cardiac arrest | 0.3 | 0.2 | 0.7 | 4.65  (2.54-8.51) |
| Cardiogenic shock | 1.6 | 1.2 | 3.2 | 2.58  (2.01-3.32) |
| Impella | 0.4 | 0.2 | 1.5 | 8.09  (4.67-14.04) |
| Intraaortic balloon | 0.5 | 0.2 | 1.6 | 8.56  (5.04-14.54) |
| Procedural AMI | 3.2 | 1.1 | 6.1 | 3.02  (2.50-3.66) |
| Vascular access complications | 4.0 | 3.0 | 8.1 | 2.26  (1.56-3.27) |
| Conversion to open heart surgery | 0.3 | 0.2 | 0.8 | 5.26  (2.92-9.48) |
| **Other complications, %** |  |  |  |  |
| Post-operative infection | 1.1 | 0.2 | 4.2 | 14.96  (10.05-22.28) |
| Respiratory complications | 8.6 | 3.8 | 26.1 | 7.66  (6.73-8.73) |
| Abbreviations: AKI acute kidney injury, AMI acute myocardial infarction, aOR adjusted odds ratio, CI confidence interval, CKD chronic kidney disease, LOS length of stay, SEM standard error of the mean. | | | | |
